# In the March 2011 issue

**DOI:** 10.1590/S1980-57642011DN05010001

**Published:** 2011

**Authors:** Ricardo Nitrini

**Affiliations:** Editor-in-Chief

**Matsuoka and Yamaguchi** reviewed the social and psychological risk factors for Alzheimer’s disease using the theory of path dependence. According to this theory, an earlier choice or event largely determines successive ones. The authors emphasized the role of education as one of these early experiences in life which determines a path that predisposes individuals to several psychosocial risk factors for dementia. They reinforced that resources for dementia prevention should be directed toward individuals with a low level of education.

**Souza-Talarico et al.** reviewed the effects of stress hormones on the brain and cognition in normal and pathological aging. Glucocorticoid receptors are mainly found in the amygdala, prefrontal cortex and the hippocampus, regions that are involved in emotional behavior, executive functions. and learning and memory. The acute and chronic effects of glucocorticoids are presented and the relevance of this area of research is emphasized by the authors. Strategies for decreasing stress levels may be beneficial for older adults.

**Anghinah et al.** presented a review on traumatic brain injury (TBI) from basic concepts of mechanisms of injury to rehabilitation. Cognitive rehabilitation in TBI is an area which calls for multidisciplinary action. TBI is a highly frequent and disabling condition that warrants further study in developing countries.

**Brucki et al.** investigated the influence of education on performance in the Mini-Mental State Examination (MMSE) using years of schooling or score on a questionnaire of literacy- the short version of the Test of Functional Health Literacy in Adults (S-TOFHLA). The authors found that both years of schooling and S-TOFHLA were strongly correlated with MMSE scores, but that S-TOFHLA had the strongest correlation with the MMSE scores.

**Wachholz and Yassuda** evaluated the capacity to understand the meaning of proverbs by elderly with different educational backgrounds. The authors also analyzed the association of the interpretation of proverbs with other cognitive functions and domains. The authors found that the capacity to interpret proverbs is strongly associated with education and performance on other tests of executive functions.

**Marques da Silva et al.** performed a systematic review of the literature to evaluate the current evidence for the use of antipsychotics in demented patients with psychotic symptoms. Their conclusion is that antipsychotics still have a place in treatment of more serious psychotic symptoms after the failure of other pharmacological and non-pharmacological treatments. The authors also concluded that new treatment alternatives are greatly needed.

**Bello and Schultz** performed a retrospective analysis of the files of 193 patients with dementia seen at an outpatient clinic of a university-associated hospital in São Paulo, Brazil. The authors found that 37 (19.17%) of the patients studied had a diagnosis consistent with a potentially reversible or preventable dementia, with head injury and alcohol-related dementias as its leading causes.

**Aramaki and Yassuda** investigated whether the gains documented after cognitive training based on metamemory and mental images were still present 18 months after the completion of the cognitive training program. Although they were only able to re-investigate approximately 45% of the original sample, the results were consistent with the maintenance of the effects of the previous training after this relatively long period of time.

**Schultz and Bertolucci** reported a case of congenital prosopagnosia in a 46-year-old woman, who also had impairment in recognizing animals. The authors reviewed the literature on this phenomenon and concluded that in the case reported, the agnosia should be classified as an associative agnosia.

**Caixeta and Lopes** reported a case of trichotillomania in an 87-year-old man with dementia due to probable Binswanger disease. The authors described trichotillomania as a poorly understood complex disorder and suggested that the pathogenesis of trichotillomania in this case may related to a defect in connectivity in the frontal-subcortical circuit.

**News and Perspectives**. In this novel section of the journal, **Sonia Maria Dozzi Brucki**, the area editor, presented a few brief reviews of relevant and recent papers to our readers.

**Ricardo Nitrini** Editor-in-Chief

